# Supplementary material for: New Class of Benzodiazepinone Derivatives as Pro-Death Agents Targeting BIR Domains in Cancer Cells
Source: Molecules. 2023 Jan 3;28(1):446. doi: 10.3390/molecules28010446 (PMC9823934; doi:10.3390/molecules28010446)
Supplement: Supplementary file 1 [file molecules-28-00446-s001.zip › molecules-2100699-SI/Supplementary Material.pdf]

Supplementary Material

New Class of Benzodiazepinone Derivatives as Pro-death Agents Targeting BIR Domains in Cancer Cells

Michele Fiore [1,+], Michele Mosconi [2,+], Francesco Bonì [2], Alice Parodi [3], Annalisa Salis [3], Bruno Tasso [4], Eloise Mastrangelo [2], Enrico Millo [3,*] and Federica Cossu [2,*]

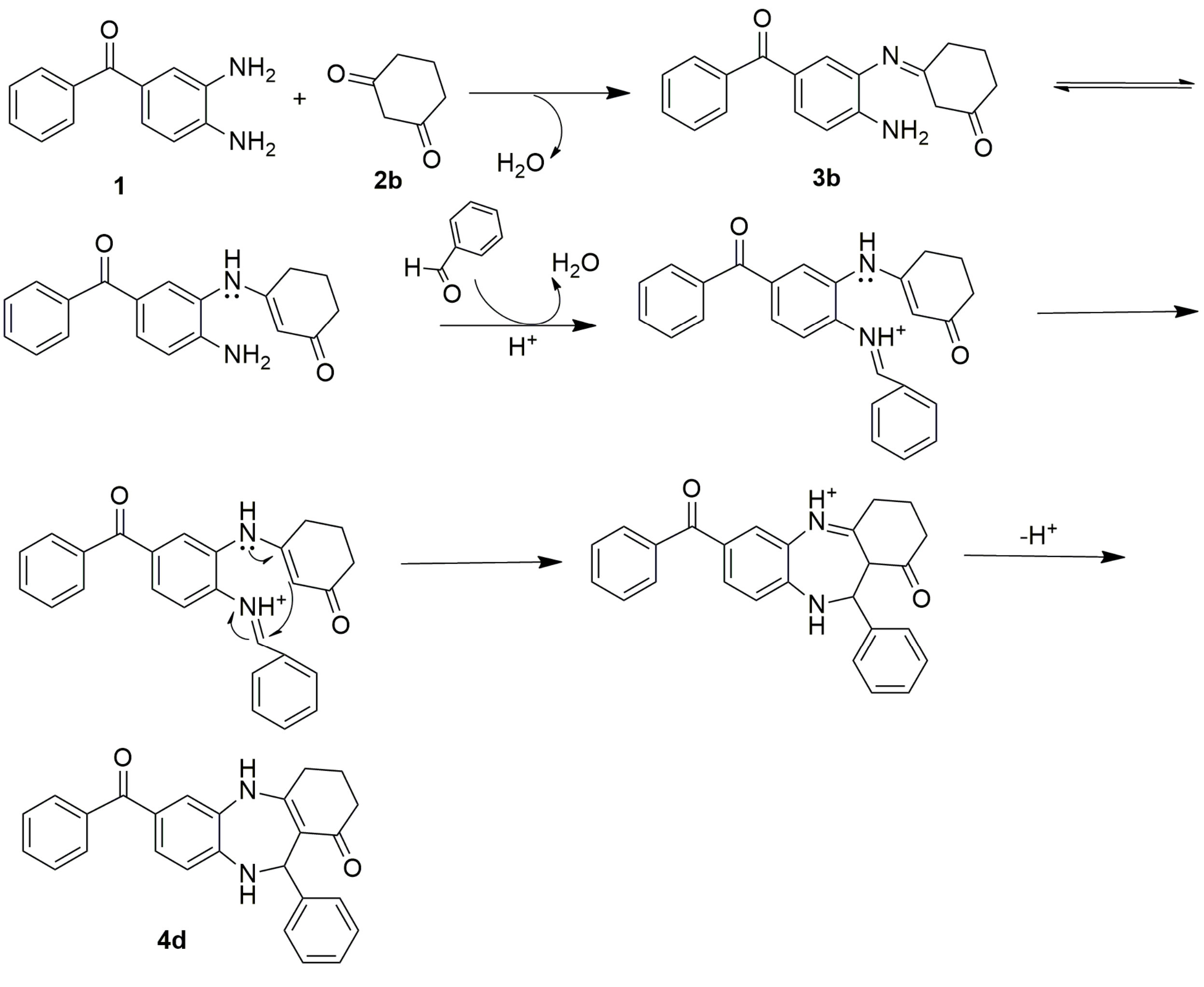

Figure S1. Plausible mechanism for the formation of 1H-dibenzo[1,4]diazepin-1-one derivatives. As example, mechanism hypothesis for forming **4d**. The reaction underwent initial nucleophilic addition and subsequent dehydration to generate intermediate **3b**. Then, the intermediate **3b** was condensed with benzaldehydes to give an intermediate, which is then converted into the final product **4d** through intramolecular cyclization.

Section S1: Synthesis of FC2-derivatives **4f-v**

*7-benzoyl-11-(4-(dimethylamino)phenyl)-2,3,4,5,10,11-hexahydro-1H-dibenzo[b,e][1,4]diazepin-1-one (**4f**)*

Compound **4f** (20.3 mg, 58%) was obtained from 4-(dimethylamino)benzaldehyde (12.0 mg, 0.08 mmol) and 3-((2-amino-5-benzoylphenyl)amino)cyclohex-2-en-1-one (25.0 mg, 0.08 mmol) in the same manner as described for **FC2,** as a yellow powder.

^{1}H NMR (400 MHz, DMSO-d_6) δ 8.86 (s, 1H, NH); 7.59-7.424 (m, 6H, arom); 7.06 (dd, J= 2.0, 8.4, 1H, arom); 7.00 (d, J=6.0, 2H, arom); 6.92 (d, J= 8.0, 1H, arom); 6.67 (d, J= 8.4, 1H, CH); 6.49 (d, J=7.8, 2H, arom); 5.66 (d, J= 6.0, 1H, NH); 2.73 (s, 6H, OCH_3); 2.70-2.53 (m, 2H, CH_2); 2.32-2.12 (m, 2H, CH_2); 1.98-1.78 (m, 2H, CH_2).

^{13}C NMR (100 MHz, DMSO-d_6) δ 194.2, 193.0, 157.2, 149.2, 144.6, 138.8, 132.4, 132.0, 130.2, 129.5, 128.8, 127.6, 125.7, 122.7, 119.8, 112.9, 112.6, 54.4, 36.5, 31.1, 21.9, 21.6.

HRMS (ESI) calculated for $C_{28}H_{28}N_3O_2$ $[M+H]^+$ 438.21814; found 438,21785.

*7-benzoyl-11-(4-methoxyphenyl)-2,3,4,5,10,11-hexahydro-1H-dibenzo[b,e][1,4]diazepin-1-one (**4g**)*

Compound **4g** (10.6 mg, 21%) was prepared from 4-methoxybenzaldehyde (18 mg, 0.12 mmol) and 3-((2-amino-5-benzoylphenyl)amino)cyclohex-2-en-1-one (36.8 mg, 0.12 mmol) in the same manner as described for **FC2**. Then, the final compound was purified by preparative HPLC to obtain the title compound as a yellow oil.

^{1}H NMR (400 MHz, DMSO-d_6) δ 9.02 (s, 1H, NH); 7.62-7.43 (m, 8H, arom); 7.23-7.21 (d, = 8.0, 2H, arom): 7.12-7.07 (m, 2H, arom); 6.67 (d, J= 8.0, 1H, CH); 5.60 (d, J= 6.4, 1H, NH); 3.68 (s, 3H, OCH_3); 2.73-2.54 (m, 2H, CH_2); 2.31-2.15 (m, 2H, CH_2); 1.98-1.75 (m, 2H, CH_2).

^{13}C NMR (100 MHz, DMSO-d_6) δ 194.3, 193.1, 156.5, 148.1, 146.2, 144.2, 141.8, 138.8, 136.4, 132.3, 130.1, 129.4, 128.7, 127.1, 125.7, 122.5, 120.4, 120.0, 112.6, 112.2, 55.1, 54.4, 36.1, 31.1, 21.9.

HRMS (ESI) calculated for $C_{27}H_{25}N_2O_3$ $[M+H]^+$ 425.18650; found 425.18555.

*7-benzoyl-11-(4-(trifluoromethyl)phenyl)-2,3,4,5,10,11-hexahydro-1H-dibenzo[b,e][1,4]diazepin-1-one (**4h**)*

Compound **4h** (17.0 mg, 37%) was prepared from 4-(trifluoromethyl)benzaldehyde (14.0 mL, 0.1 mmol) and 3-((2-amino-5-benzoylphenyl)amino)cyclohex-2-en-1-one (30.5 mg, 0.1 mmol) in the same manner as described for **FC2.**

Then, the final compound was purified by preparative HPLC to obtain the title compound as a yellow oil.

^{1}H NMR (400 MHz, DMSO-d_6) δ 9.04 (s, 1H, NH); 7.67-7.42 (m, 8H, arom); 7.31 (d, J= 8.4, 2H arom); 7.16-7.09 (m, 2H, arom); 6.69 (d, J= 8.4, 1H, CH); 5.81 (d, J= 5.8, 1H, NH); 2.76-2.57 (m, 2H, CH_2); 2.34-2.16 (m, 2H, CH_2); 2.00-1.79 (m, 2H, CH_2).

^{13}C NMR (100 MHz, DMSO-d_6) δ 194.3, 193.3, 157.8, 149.6, 143.5, 138.7, 132.1, 130.1, 129.5, 128.8, 128.3, 128.3, 127.5, 127.2, 126.1, 126.0, 125.6, 125.6, 125.6, 123.4, 123.1, 120.0, 111.5, 55.1, 36.4, 31.1, 21.8.

HRMS (ESI) calculated for $C_{27}H_{22}F_3N_2O_2[M+H]^+$ 463.16332; found 463.16296.

*7-benzoyl-11-(4-chlorophenyl)-2,3,4,5,10,11-hexahydro-1H-dibenzo[b,e][1,4]diazepin-1-one (**4i**)*

Compound **4i** (10.5 mg, 10%) was prepared from 4-chlorobenzaldehyde (36 mg, 0.24 mmol) and 3-((2-amino-5-benzoylphenyl)amino)cyclohex-2-en-1-one (77.6 mg, 0.24 mmol) in the same manner as described for **FC2**.Then, the final compound was purified by preparative HPLC to obtain the title compound as a yellow oil.

^{1}H NMR (400 MHz, DMSO-d$_6$) δ 9.03 (s, 1H, NH); 7.67-7.41 (m, 8H, arom); 7.32 (d, J= 8.0, 2H, arom); 7.15-7.08 (m, 2H, arom); 6.68 (d, J= 8.4, 1H, CH); 5.79 (d, J= 6.0, 1H, NH); 2.75-2.57 (m, 2H, CH$_2$); 2.32-2.14 (m, 2H, CH$_2$); 2.02-1.79 (m, 2H, CH$_2$).

^{13}C NMR (100 MHz, DMSO-d$_6$) δ 194.3, 193.0, 157.1, 148.2, 146.8, 138.9, 135.6, 134.5, 132.3, 130.1, 129.5, 128.8, 126.3, 125.7, 122.4, 120.2, 120.0, 112.8, 112.3, 55.2, 35.9, 31.1, 21.9.

HRMS (ESI) calculated for $C_{26}H_{22}ClN_2O_2$[M+H]$^+$ 429.13697; found 429.13603.

*7-benzoyl-11-(4-hydroxyphenyl)-2,3,4,5,10,11-hexahydro-1H-dibenzo[b,e][1,4]diazepin-1-one **(4j)***

Compound **4j** (10.5 mg, 25 %) was obtained from 4-hydroxybenzaldehyde (13.0 mg, 0.09 mmol) and 3-((2-amino-5-benzoylphenyl)amino)cyclohex-2-en-1-one (26.5 mg, 0.09 mmol) in the same manner as described for **FC2**, as a yellow powder.

^{1}H NMR (400 MHz, DMSO-d$_6$) δ 9.04 (s, 1H, NH); 8.63 (s, 1H, OH); 7.62-7.43 (m, 8H, arom); 7.24 (d, J=8.4, 2H, arom); 7.08 (m, 2H, arom); 6.66 (d, J= 46.0, 1H, CH); 5.60 (d, J= 46.0, 1H, NH); 2.74-2.57 (m, 2H, CH$_2$); 2.30-2.15 (m, 2H, CH$_2$); 1.97-1.74 (m, 2H, CH$_2$).

^{13}C NMR (100 MHz, DMSO-d$_6$) δ 194.3, 193.0, 156.9, 148.3, 145.8, 143.4, 141.8, 138.8, 135.9, 132.2, 130.1, 129.7, 128.8, 127.0, 125.7, 122.4, 120.8, 120.0, 113.1, 112.2, 55.1, 36.1, 31.1, 21.8.

HRMS (ESI) calculated for $C_{26}H_{22}N_3O_5$ [M+H]$^+$ 456.15593; found 456.15588.

*7-benzoyl-11-(3-methoxyphenyl)-2,3,4,5,10,11-hexahydro-1H-dibenzo[b,e][1,4]diazepin-1-one **(4k)***

Compound **4k** (11 mg, 13%) was prepared from 3-methoxybenzaldehyde (26.8 mL, 0.2 mmol) and 3-((2-amino-5-benzoylphenyl)amino)cyclohex-2-en-1-one (61 mg, 0.2 mmol) in the same manner as described for **FC2**. Then, the final compound was crystallized in acetonitrile to obtain the pure compound as a brown powder.

^{1}H NMR (400 MHz, DMSO-d$_6$) δ 8.88 (s, 1H, NH); 7.58-7.39 (m, 8H, arom); 7.14 (dd, J= 2.0, 8.4, 1H, arom); 7.06 (dd, J= 1.6, 8.0, 1H, arom); 6.72 (d, J=8.0, 2H, arom); 6.67 (d, J= 6.0, 1H, CH); 5.79 (d, J= 46.0, 1H, NH); 3.61 (s, 3H, OCH$_3$); 2.75-2.62 (m, 2H, CH$_2$); 2.31-2.16 (m, 2H, CH$_2$); 1.97-1.75 (m, 2H, CH$_2$).

^{13}C NMR (100 MHz, DMSO-d$_6$) δ 194.3, 193.0, 157.1, 148.3, 147.2, 141.9, 138.3, 135.3, 132.6, 130.1, 129.5, 128.9, 128.0, 126.1, 123.0, 122.4, 121.8, 120.1, 56.9, 55.2, 36.4, 31.1, 21.9.

HRMS (ESI) calculated for $C_{27}H_{26}N_2O_3$ [M+H]$^+$ 425.18650; found 438.18555.

7-benzoyl-11-(3-hydroxyphenyl)-2,3,4,5,10,11-hexahydro-1H-dibenzo[b,e][1,4]diazepin-1-one **(4l)**

Compound **4l** (14.0 mg, 46%) was prepared from 3-hydroxybenzaldehyde (9.5 mg, 0.08 mmol) and 3-((2-amino-5-benzoylphenyl)amino)cyclohex-2-en-1-one (23.0 mg, 0.08 mmol) in the same manner as described for **FC2**. Then, the final compound was purified by preparative HPLC to obtain the title compound as a yellow oil.

^{1}H NMR (400 MHz, DMSO-d_6) δ 8.87 (s, 1H, NH); 8.64 (s, 1, OH); 7.54-7.37 (m, 8H, arom); 7.12 (dd, J= 2.0, 8.0, 1H, arom); 7.04 (d, J= 8.0, 1H, arom); 6.77 (d, J= 8.0, 2H, arom); 6.66 (d, J= 6.4, 1H, CH); 5.77 (d, J= 6.0, 1H, NH); 2.73-2.61 (m, 2H, CH_2); 2.30-2.15 (m, 2H, CH_2); 1.98-1.74 (m, 2H, CH_2).

^{13}C NMR (100 MHz, DMSO-d_6) δ 194.3, 193.0, 156.4, 148.1, 147.3, 143.5, 138.2, 135.6, 132.3, 130.1, 129.7, 128.8, 128.1, 125.7, 123.1, 122.5, 121.7, 120.1, 55.1, 36.4, 31.1, 21.9.

HRMS (ESI) calculated for $C_{26}H_{23}N_2O_3$ $[M+H]^+$ 411.17085; found 411.17075.

*7-benzoyl-11-(3-nitrophenyl)-2,3,4,5,10,11-hexahydro-1H-dibenzo[b,e][1,4]diazepin-1-one **(4m)***

Compound **4m** (8.0 mg, 9%) was obtained from 3-nitrobenzaldehyde (30.2 mg, 0.2 mmol) and 3-((2-amino-5-benzoylphenyl)amino)cyclohex-2-en-1-one (61.2 mg, 0.2 mmol) in the same manner as described for **FC2**, as a yellow powder.

^{1}H NMR (400 MHz, DMSO-d_6) δ 9.09 (s, 1H, NH); 8.03 (t, J=2.0, 1H, arom); 7.94-7.89 (m, 1H, arom); 7.63-7.41 (m, 8H, arom); 7.18 (d, J= 6.4, 1H, arom); 7.10 (dd, J= 2.4, 8.8, 1H arom); 6.70 (d, J= 8.8, 1H, CH); 5.85 (d, J= 6.0, 1H, NH); 2.77-2.63 (m, 2H, CH_2); 2.32-2.18 (m, 2H, CH_2); 2.02-1.78 (m, 2H, CH_2).

^{13}C NMR (100 MHz, DMSO-d_6) δ 194.3, 193.3, 158.0, 148.3, 147.1, 143.3, 138.6, 134.0, 132.2, 130.2, 129.5, 128.8, 128.6, 126.0, 123.1, 122.5, 121.9, 120.1, 55.1, 36.3, 31.1, 21.8.

HRMS (ESI) calculated for $C_{26}H_{22}N_3O_4$ $[M+H]^+$ 440.16102; found 440.16050.

*11-(3-aminophenyl)-7-benzoyl-2,3,4,5,10,11-hexahydro-1H-dibenzo[b,e][1,4]diazepin-1-one (**4n**)*

Compound **4n** (9 mg, 6%) was prepared from 3-aminobenzaldehyde (42.5 mg, 0.35 mmol) and 3-((2-amino-5-benzoylphenyl)amino)cyclohex-2-en-1-one (110.1 mg, 0.35 mmol) in the same manner as described for **FC2.** Then, the final compound was purified by preparative HPLC to obtain the title compound as a yellow oil.

^{1}H NMR (400 MHz, DMSO-d_6) δ 8.82 (s, 1H, NH); 7.59-7.38 (m, 8H, arom); 7.16 (dd, J= 2.0, 8.4, 1H arom); 7.06 (dd, J= 1.6, 8.0, 1H, arom); 6.75 (d, J= 8.0, 2H, arom); 6.67 (d, J= 6.4, 1H, CH); 5.78 (d, J= 6.0, 1H, NH); 5.53 (s, 2H, NH_2); 2.71-2.58 (m, 2H, CH_2); 2.27-2.11 (m, 2H, CH_2); 1.95-1.72 (m, 2H, CH_2).

^{13}C NMR (100 MHz, DMSO-d_6) δ 194.3, 193.2, 148,6 148.3, 147.0, 143.5, 138.3, 134.5, 132.2, 130.2, 129.4, 128.8, 127.9, 126.1, 123.2, 122.4, 121.8, 120.0, 55.1, 36.3, 31.1, 21.8.

HRMS (ESI) calculated for $C_{26}H_{24}N_3O_2$ $[M+H]^+$ 410.18684; found 410.18591.

*7-benzoyl-11-(3,4-dimethoxyphenyl)-2,3,4,5,10,11-hexahydro-1H-dibenzo[b,e][1,4]diazepin-1-one (**4o**)*

Compound **4o** (8 mg, 11%) was prepared from 3,4-dimethoxybenzaldehyde (30 mg, 0.18 mmol) and 3-((2-amino-5-benzoylphenyl)amino)cyclohex-2-en-1-one (58 mg, 0.18 mmol) in the same manner as described for

FC2. Then, the final compound was purified by preparative HPLC to obtain the title compound as a yellow oil.

^{1}H NMR (400 MHz, DMSO-d$_6$) δ 8.87 (s, 1H, NH); 7.60-7.45 (m, 6H, arom); 7.07 (dd, J= 2.0, 8.4, 1H, arom); 6.99 (d, J=6.4, 1H, arom); 6.67 (d, J= 8.0, 2H, arom); 6.56 (d, J=8.0, 1H, CH); 6.54-6.46 (m, 1H, arom); 5.61 (d, J= 6.0, 1H, NH); 3.59 (s, 3H, OCH$_3$); 3.33 (s, 3H, OCH$_3$); 2.71-2.54 (m, 2H, CH$_2$); 2.30-2.14 (m, 2H, CH$_2$); 1.96-1.75 (m, 2H, CH$_2$).

^{13}C NMR (100 MHz, DMSO-d$_6$) δ 194.3, 193.0, 156.6, 148.0, 146.2, 142.2, 141.8, 138.8, 136.3, 132.5, 130.1, 129.2, 128.7, 127.0, 125.6, 122.6, 120.3, 118.9, 112.5, 112.1, 55.9, 55.1, 54.7, 36.1, 31.1, 21.8.

HRMS (ESI) calculated for $C_{28}H_{27}N_2O_4$ [M+H]$^+$ 455.19707; found 455.19695.

7-benzoyl-11-(3,4-dihydroxyphenyl)-2,3,4,5,10,11-hexahydro-1H-dibenzo[b,e][1,4]diazepin-1-one ***(4p)***

Compound **4p** (7.0 mg, 27%) was prepared from 3,4-dihydroxybenzaldehyde (8.3 mg, 0.06 mmol) and 3-((2-amino-5-benzoylphenyl)amino)cyclohex-2-en-1-one (18.5 mg, 0.06 mmol) in the same manner as described for **FC2.**

Then, the final compound was purified by preparative HPLC to obtain the title compound as a yellow oil.

^{1}H NMR (400 MHz, DMSO-d$_6$) δ 8.91 (s, 1H, NH); 8.77 (s, 1H, OH); 8.64 (s, 1H, OH); 7.58-7.40 (m, 6H, arom); 7.08 (dd, J= 2.0, 8.0, 1H, arom); 6.96 (d, J=6.6 1H, arom); 6.67 (d, J= 8.0, 2H, arom); 6.54 (d, J=6.4 1H, CH); 6.51-6.43 (m, 1H, arom); 5.58 (d, J= 4.0, 1H, NH); 2.69-2.52 (m, 2H, CH$_2$); 2.28-2.12 (m, 2H, CH$_2$); 1.96-1.73 (m, 2H, CH$_2$).

^{13}C NMR (100 MHz, DMSO-d$_6$) δ 194.3, 193.0, 157.2, 148.1, 145.6, 145.3, 138.9, 135.2, 132.1, 130.2, 129.8, 128.8, 127.7, 122.6, 120.1, 119.6, 118.5, 112.4, 111.9, 55.3, 36.5, 31.1, 21.8.

HRMS (ESI) calculated for $C_{26}H_{23}N_2O_4$ [M+H]$^+$ 427.16577; found 427.16489.

7-benzoyl-11-(2,2-difluorobenzo[d][1,3]dioxol-5-yl)-2,3,4,5,10,11-hexahydro-1H dibenzo[b,e][1,4]diazepin-1-one (***4q***)

Compound **4q** (10.8 mg, 16%) was prepared from 2,2-difluorobenzo[*d*][1,3]dioxole-5-carbaldehyde (36.8 mg, 0.2 mmol) and 3-((2-amino-5-benzoylphenyl)amino)cyclohex-2-en-1-one (61 mg, 0.2 mmol) in the same manner as described for **FC2.** Then, the final compound was purified by preparative HPLC to obtain the title compound as a yellow oil.

^{1}H NMR (400 MHz, DMSO-d$_6$) δ 8.89 (s, 1H, NH); 7.62-7.44 (m, 6H, arom); 7.06 (dd, J= 2.0, 8.4, 1H arom); 6.98 (d, 6.8, 1H, arom); 6.67 (d, J= 8.4, 2H, arom); 6.60 (d, J=8.0, 1H, CH); 6.55-6.47 (m, 1H, arom); 5.59 (d, J= 6.0, 1H, NH); 2.70-2.54 (m, 2H, CH$_2$); 2.28-2.12 (m, 2H, CH$_2$); 1.98-1.73 (m, 2H, CH$_2$).

^{13}C NMR (100 MHz, DMSO-d$_6$) δ 194.3, 193.0, 179.4, 156.2, 148.7, 145.8, 143.7, 141.9, 138.8, 136.3, 132.7, 130.3, 129.2, 128.8, 127.1, 125.5, 122.5, 120.1, 119.3, 112.4, 112.1, 55.2, 36.1, 31.1, 22.1.

HRMS (ESI) calculated for $C_{27}H_{21}F_2N_2O_4$ [M+H]$^+$ 475.14693; found 475.14607.

7-benzoyl-11-(4-hydroxy-3-methoxyphenyl)-2,3,4,5,10,11-hexahydro-1Hdibenzo[b,e][1,4]diazepin-1-one (***4r)***

Compound **4r** (19.7 mg, 56%) was prepared from 4-hydroxy-3-methoxybenzaldehyde (12.2 mg, 0.08 mmol) and 3-((2-amino-5-benzoylphenyl)amino)cyclohex-2-en-1-one (25.1 mg, 0.08 mmol) in the same manner as described for **FC2.** Then, the final compound was crystallized in acetonitrile to obtain the pure compound as a brown powder.

^{1}H NMR (400 MHz, DMSO-d$_6$) δ 8.88 (s, 1H, NH); 8.64 (s, 1H, OH); 7.58-7.44 (m, 6H, arom); 7.07 (dd, J= 2.0, 8.4, 1H, arom); 6.96 (d, J= 6.8, 1H, arom); 6.81 (d, J=1.6, 1H, arom); 6.69 (d, J= 8.4, 1H, CH); 6.47 (d, J=7.6, 1H, arom); 6.38 (d, J=6.6, 1H, arom); 5.67 (d, J= 6.0, 1H, NH); 3.30 (s, 3H, OCH_3); 2.72-2.53 (m, 2H, CH_2); 2.31-2.15 (m, 2H, CH_2); 1.97-1.80 (m, 2H, CH_2).

^{13}C NMR (100 MHz, DMSO-d$_6$) δ 194.2, 193.0, 157.4, 147.7, 145.3, 144.6, 138.8, 135.6, 132.0, 130.4, 129.5, 128.8, 127.9, 125.7, 122.7, 120.0, 119.5, 118.6, 112.5, 112.3, 55.9, 54.9, 36.5, 31.1, 21.9.

HRMS (ESI) calculated for $C_{27}H_{25}N_2O_4$ [M+H]$^+$ 441.18142; found 441.18060.

*7-benzoyl-11-(3-hydroxy-4-methoxyphenyl)-2,3,4,5,10,11-hexahydro-1Hdibenzo[b,e][1,4]diazepin-1-one (**4s**)*

Compound **4s** (26.0 mg, 65%) was prepared from 3-hydroxy-4-methoxybenzaldehyde (14.5 mg, 0,1 mmol) and 3-((2-amino-5-benzoylphenyl)amino)cyclohex-2-en-1-one (30.5 mg, 0.1 mmol) in the same manner as described for **FC2.** Then, the final compound was purified by preparative HPLC to obtain the title compound as a yellow oil.

^{1}H NMR (400 MHz, DMSO-d$_6$) δ 8.88 (s, 1H, NH); 8.65 (s, 1H, OH); 7.60-7.44 (m, 6H, arom); 7.08 (dd, J= 2.0, 8.4, 1H arom); 6.99 (d, J=6.4, 1H, arom); 6.66 (d, J= 6.0, 2H, arom); 6.63 (d, J= 6.4, 1H, CH); 6.57-6.48 (m, 1H, arom); 5.62 (d, J= 4.0, 1H, NH); 3.61 (s, 3H, OCH_3); 2.72-2.55 (m, 2H, CH_2); 2.31-2.14 (m, 2H, CH_2); 1.99-1.76 (m, 2H, CH_2).

^{13}C NMR (100 MHz, DMSO-d$_6$) δ 194.2, 193.0, 157.3, 146.4, 146.3, 144.4, 138.8, 137.5, 132.0, 130.1, 129.7, 129.5, 128.8, 127.7, 125.8, 122.8, 119.8, 118.2, 115.3, 112.8, 112.1, 55.9, 54.5, 36.5, 31.1, 21.8.

HRMS (ESI) calculated for $C_{27}H_{25}N_2O_4$ [M+H]$^+$ 441.18142; found 441.18060.

*7-benzoyl-11-(2-hydroxy-3-methoxyphenyl)-2,3,4,5,10,11-hexahydro-1Hdibenzo[b,e][1,4]diazepin-1-one (**4t**)*

Compound **4t** (21.2 mg, 55%) was obtained from 2-hydroxy-3-methoxybenzaldehyde (13.0 mg, 0.09 mmol) and 3-((2-amino-5-benzoylphenyl)amino)cyclohex-2-en-1-one (26.5 mg, 0.09 mmol) in the same manner as described for **FC2**, as a yellow powder.

^{1}H NMR (400 MHz, DMSO-d$_6$) δ 9.02 (s, 1H, NH); 8.94 (s, 1H, OH); 7.68-7.40 (m, 6H, arom); 7.02 (dd, J=2.0, 8.4, 1H, arom); 6.67 (d, J= 8.4, 1H, arom); 6.57 (d, J=8.0, 1H, CH); 6.42 (t, J=7.8, 1H, arom); 6.27 (dd, J=1.6, 8.0, 1H, arom); 6.07 (d, J=6.0, 1H, arom); 5.97 (d, J= 6.4, 1H, NH); 3.72 (s, 3H, OCH_3); 2.80-2.57 (m, 2H, CH_2); 2.30-2.12 (m, 2H, CH_2); 1.97-1.82 (m, 2H, CH_2).

^{13}C NMR (100 MHz, DMSO-d$_6$) δ 194.3, 193.2, 157.8, 147.9, 144.5, 143.9, 138.7, 132.1, 130.6, 130.5, 129.5, 128.8, 128.5, 125.6, 122.7, 120.3, 118.9, 118.3, 111.1, 111.0, 56.2, 52.1, 36.5, 31.2, 22.0.

HRMS (ESI) calculated for $C_{27}H_{25}N_2O_4$ [M+H]$^+$ 441.18142; found 441.18058.

*7-benzoyl-11-(4-hydroxy-3-nitrophenyl)-2,3,4,5,10,11-hexahydro-1H-dibenzo[b,e][1,4]diazepin-1-one (**4u**)*

Compound **4u** (12.5 mg, 30%) was obtained from 4-hydroxy-3-nitrobenzaldehyde (16.7 mg, 0.1 mmol) and 3-((2-amino-5-benzoylphenyl)amino)cyclohex-2-en-1-one (30.5 mg, 0.1 mmol) in the same manner as described for **FC2**, as a yellow powder.

^{1}H NMR (400 MHz, DMSO-d_6) δ 9.07 (s, 1H, NH); 8.72 (s, 1H, OH); 8.57 (s, 1H arom); 7.61-7.48 (m, 6H, arom); 7.18 (d, J= 8.0, 1H, arom); 7.09 (dd, J= 2.0, 8.0, 2H, arom); 6.98 (d, J=6.4, 1H, arom); 6.70 (d, J= 8.0, 1H, CH); 5.84 (d, J= 6.0, 1H, NH); 2.78-2.64 (m, 2H, CH_2); 2.31-2.17 (m, 2H, CH_2); 2.02-1.77 (m, 2H, CH_2).

^{13}C NMR (100 MHz, DMSO-d_6) δ 194.3, 193.1, 158.9, 148.5, 144.7, 141.3, 138.8, 135.3, 132.2, 130.1, 129.7, 128.8, 127.4, 122.5, 120.3, 119.2, 118.5, 112.3, 112.0, 55.1, 36.4, 31.1, 21.9.

HRMS (ESI) calculated for $C_{26}H_{22}N_2O_3$ $[M+H]^+$ 411.17085; found 411.17050.

*7-benzoyl-11-(4-hydroxy-3-methoxy-5-nitrophenyl)-2,3,4,5,10,11-hexahydro1H dibenzo[b,e][1,4]diazepin-1-one (**4v**)*

Compound **4v** (10 mg, 20%) was obtained from 4-hydroxy-3-methoxy-5-nitrobenzaldehyde (19.8 mg, 0.1 mmol) and 3-((2-amino-5-benzoylphenyl)amino)cyclohex-2-en-1-one (30.5 mg, 0.1 mmol) in the same manner as described for **FC2**, as a yellow powder.

^{1}H NMR (400 MHz, DMSO-d_6) δ 9.04 (s, 1H, NH); 8.80 (s, 1H, OH); 8.43 (s, 1H, arom); 7.59-7.42 (m, 6H, arom); 7.16 (dd, J= 1.6 8.4, 1H, arom); 7.07 (dd, J= 2.0, 8.0, 2H, arom); 6.69 (d, J= 8.0, 1H, CH); 5.81 (d, J= 6.2, 1H, NH); 3.59 (s, 3H, OCH_3); 2.75-2.62 (m, 2H, CH_2); 2.29-2.15 (m, 2H, CH_2); 1.98-1.73 (m, 2H, CH_2).

^{13}C NMR (100 MHz, DMSO-d_6) δ 194.4, 193.0, 158.7, 148.3, 145.3, 144.7, 141.2, 138.7, 135.2, 132.1, 130.1, 129.6, 128.8, 127.5, 122.5, 120.4, 120.1, 118.5, 112.5, 112.2, 55.1, 54.9, 36.4, 31.1, 21.9.

HRMS (ESI) calculated for $C_{27}H_{24}N_3O_6$ $[M+H]^+$ 486.16650; found 486.16637.
