# Supplementary figures and images for: New Class of Benzodiazepinone Derivatives as Pro-Death Agents Targeting BIR Domains in Cancer Cells
Source: Molecules. 2023 Jan 3;28(1):446. doi: 10.3390/molecules28010446 (PMC9823934; doi:10.3390/molecules28010446)

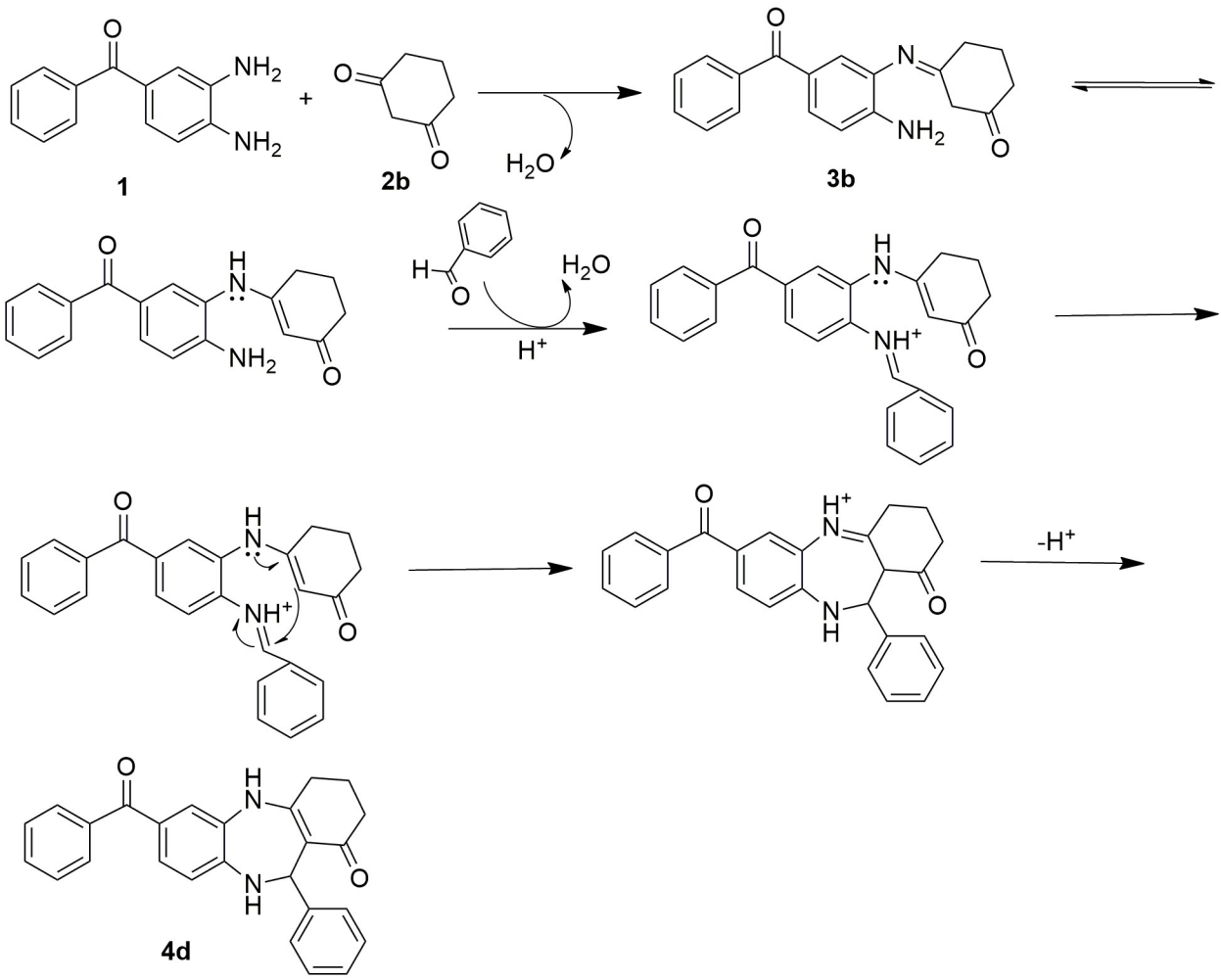

Supplement: Supplementary file 1 [file molecules-28-00446-s001.zip › molecules-2100699-SI/Scheme S1.pdf]
